# *Angelica Dahurica* ethanolic extract improves impaired wound healing by activating angiogenesis in diabetes

**DOI:** 10.1371/journal.pone.0177862

**Published:** 2017-05-24

**Authors:** Xiao-na Zhang, Ze-jun Ma, Ying Wang, Bei Sun, Xin Guo, Cong-qing Pan, Li-ming Chen

**Affiliations:** 1 From the Department of Endocrinology and Metabolism, Tianjin Medical University General Hospital, Tianjin, People’s Republic of China; 2 From 2011 Collaborative Innovation Center of Tianjin for Medical Epigenetics, Key Laboratory of Hormone and Development, Ministry of Health, Metabolic Disease Hospital and Tianjin Institute of Endocrinology, Tianjin Medical University, Tianjin, People’s Republic of China; University of Edinburgh, UNITED KINGDOM

## Abstract

Abnormal angiogenesis plays an important role in impaired wound healing and development of chronic wounds in diabetes mellitus. *Angelica dahurica* radix is a common traditional Chinese medicine with wide spectrum medicinal effects. In this study, we analyzed the potential roles of *Angelica dahurica* ethanolic extract (ADEE) in correcting impaired angiogenesis and delayed wound healing in diabetes by using streptozotocin-induced diabetic rats. ADEE treatment accelerated diabetic wound healing through inducing angiogenesis and granulation tissue formation. The angiogenic property of ADEE was subsequently verified *ex vivo* using aortic ring assays. Furthermore, we investigated the *in vitro* angiogenic activity of ADEE and its underlying mechanisms using human umbilical vein endothelial cells. ADEE treatment induced HUVECs proliferation, migration, and tube formation, which are typical phenomena of angiogenesis, in dose-dependent manners. These effects were associated with activation of angiogenic signal modulators, including extracellular signal-regulated kinase 1/2 (ERK1/2), Akt, endothelial nitric oxide synthase (eNOS) as well as increased NO production, and independent of affecting VEGF expression. ADEE-induced angiogenic events were inhibited by the MEK inhibitor PD98059, the PI3K inhibitor Wortmannin, and the eNOS inhibitor L-NAME. Our findings highlight an angiogenic role of ADEE and its ability to protect against impaired wound healing, which may be developed as a promising therapy for impaired angiogenesis and delayed wound healing in diabetes.

## Introduction

Impaired wound healing associated with diabetes results in serious diabetic complications, such as chronic open wounds, amputation, or even death [1, 2]. Wound healing is a dynamic and sequential process involving coagulation, inflammation, angiogenesis, tissue formation, and tissue remodeling [3, 4].

Angiogenesis, the formation of new blood vessels from preexisting vessels, is a crucial process for wound healing [5], which is seriously undermined in diabetic wounds [6, 7]. Since the indispensable oxygen and blood supply for regenerating new tissues can not be supplied timely from the new vessels, diabetic wounds are impossible to heal. Endothelial cell migration, proliferation, and tube formation are essential processes in angiogenesis [8]. These angiogenic processes rely on the activation of multiple signaling pathways in endothelial cells by endogenous or exogenous angiogenic substances, involving extracellular signal- regulated kinase 1/2 (ERK1/2) [9], Akt [10], endothelial nitric oxide synthase (eNOS), and endothelial nitric oxide (NO) production [11].

Prolonged inflammation is another hallmark of diabetic non-healing wounds [12]. Mounting evidence have shown that compared with non-diabetic wounds, diabetic wounds has more and prolonged infiltration of inflammatory cells and expression of inflammatory factors [13]. Sustained inflammatory response creates a protease (neutrophil elastase, matrix Metalloproteinases, and gelatinase) rich hostile microenvironment [14], resulting in degradation of extracellular matrix and growth factors, which significantly delays the healing.

The usage of herbal medicines in wound management dates back centuries ago and remains popular today because of their multifaceted activities and minimal unwanted side effects. In recent years, great progress has been made in demonstrating the potential of plant derived medicines in wound healing and clarifying those underlying mechanisms (reviewed in [15]). Among those herbs, *Angelica sinensis***, *a*** well-known species of *Angelica*, has been found to stimulate wound healing and increase the strength of the healed wounds [16]. In traditional Chinese medicine, another wildly-grown *Angelica* species named *Angelica dahurica (Baizhi in Chinese)*, is used as an herb that purge the body of any negative influences such as heat, clamminess, dryness, and cold on the skin[17]. A number of active constituents such as imperatorin, isoimperatorin, oxypeucedanin, byakangelicol, and byakangelicin have been identified from *Angelica Dahurica Radix* [18]. *Angelica dahurica* and its constituents have been reported to possess wide spectrum pharmacological actions. The ethanolic extract of *Angelica Dahurica Radix* was reported to promote adhesion and migration in melanocytes [19]. Imperatorin and isoimperatorin, two major constituents from ethanolic extract of *Angelica Dahurica Radix*, induces vasodilation via regulating NO synthesis in endothelium [20]. And imperatorin attenuates cardiac hypertrophy through significantly activating phosphorylation of eNOS and preventing the decreased NO production in transverse aortic constriction mice [21]. Moreover, imperatorin protected cerebral cells against ischemia-induced apoptosis through activating ERK pathways [22]. Other activities of *Angelica Dahurica* such as antioxidant [23] and anti-inflammatory [24] were also widely reported.

In traditional Chinese medicine, *Angelica dahurica* has been included in a number of formulae. Particularly, a previous study [25]by our group showed the wound healing effects of a TCM formula named Tuo-Li-Xiao-Du-San (TLXDS), which is composed of four herbs: *Angelica dahurica* (Bai Zhi), *Angelica sinensis* (Dang Gui), *Astragalus membranaceus* (Huang Qi), and *thorns of Gleditsia sinensis* (Zao Jiao Ci), in STZ-induced diabetic rats. We observed that compared with control, TLXDS-treated diabetic rats had more new vessels formed in the proliferative phase and accelerated inflammation resolution. Considering its multifaceted effects, we speculated that *Angelica Dahurica* might play a positive role in diabetic wound healing through inducing angiogenesis and reducing inflammation. To testify our speculation, we examined the therapeutic effects of *Angelica dahurica* ethanolic extract (ADEE) on excisional cutaneous wound repair using STZ-induced diabetic rats, and We further checked the angiogenic effects of ADEE and the underlying mechanisms through the activation of ERK1/2, Akt, eNOS, and NO production using isolated rat aortic ring and human umbilical vein endothelial cells (HUVECs). In addition, we demonstrated that ADEE accelerated inflammation resolution in diabetic wounds.

## Materials and methods

### Reagents and chemicals

*Angelica dahurica* radix was obtained from and authenticated by TASLY Pharmaceutical Group Co. ltd. (Tianjin, China). *Angelica dahurica* 70% ethanolic extract (ADEE) was prepared and packed by the department of Pharmaceutical Sciences, Tianjin University of Traditional Chinese Medicine (Tianjin, China) using standardized procedure. Briefly, the crude herb was powdered and then extracted with 70% ethanol for three times. The extracts were gathered and filtered to remove the insoluble remnant. Solvents were removed through freeze-drying. The condensate was stored at– 20°C. The extraction yield of *Angelica dahurica* was approximately 18% (w/w). The chemical profile of ADEE was detected by high performance liquid chromatography (HPLC) method using imperatorin as a reference standard. Chromatogram of imperatorin and imperatorin contents in ADEE was shown in S1A–S1C Fig.

In animal studies, the extracts were freshly prepared for oral gavage to a concentration of 20% by diluting with sterile water. For cell experiments, the extracts were diluted with Dulbecco's modified Eagle's medium (DMEM; Gibco) to a range of concentration from 10 to 400 μg/ml.

Recombinant human VEGF was purchased from Sciencell research laboratories (San Diego, CA). Matrigel was obtained from BD Biosciences (Frankin Lakes, NJ, U.S.A.). Antibody for VEGF was obtained from Abcam (Massachusetts, US). Antibodies for total ERK, Akt, eNOS and phospho-ERK (Thr-202/Tyr-204), phospho-Akt (Ser473), and phospho-eNOS (Ser113) were obtained from Cell Signaling Technology (Beverly, MA, U.S.A.). PD98059, wortmannin, and L-NG-nitroarginine methyl ester (L-NAME) were purchased from Selleck Chemicals (Houston, TX). All other reagents were purchased from Sigma (St. Louis, MO, U.S.A.) unless indicated otherwise.

### Animals and cell culture

All efforts were made to minimize animal suffering and to reduce the number of animals. Sprague Dawley rats (290±10g) were purchased from Beijing HFK Bioscience CO., LTD (Beijing, China). Animals were raised under specific-pathogen-free conditions at the Chinese Academy of medical Sciences &Peking Union Medical College Institute of Biomedical Engineering Animal SPF facility (Tianjin, China). Rats were maintained under conditions of controlled temperature (22–25°C) and humidity (50%-60%), on a 12 h light/dark cycle (lights on, 6 a.m. to 6 p.m.), with free access to food and ordinary chow. To sacrifice the animals, rats were euthanized with an overdose intraperitoneal injection of sodium pentobarbital (200 mg/kg). This study was approved by Experimental Animal Ethical Committee of Tianjin Medical University, and all procedures with animals complied with rules of the Guide for the Care and Use of Laboratory Animals of the National Institutes of Health as well as the guidelines of the Animal Welfare Act.

EA.hy926 endothelial cells, a human umbilical vein endothelial cell line, were obtained from the Cell Resource Center of the Shanghai Institute of Life Science, Chinese Academy of Sciences, China. EA.hy926 cells were cultured in low-glucose Dulbecco's modified Eagle's medium (DMEM; Gibco) supplemented with 10% fetal bovine serum (FBS; Sciencell, San Diego, CA) in a humidified atmosphere with 5% CO_2_ at 37°C.

### Induction of diabetes

Diabetes was induced in rats by a single intravenous injection of STZ (Sigma-Aldrich, St. Louis, MO, USA) at a dose of 50 mg/kg body weight in 0.1 mol/L citrate-phosphate buffer, pH 4.5. Control rats were injected with vehicle alone. Tail vein blood glucose was measured with an Accu-Chek Aviva glucometer (Roche Diagnostics GmbH, Germany). Rats with blood glucose levels ≥16.7 mmol/L were considered diabetic.

### Cutaneous excision wound model and ADEE treatment

A rat model of cutaneous excisional wound healing was used [26]. Surgical procedures were performed under proper anesthesia and all necessary steps to minimize suffering were taken. Briefly, four weeks after STZ injection, all rats were anesthetized with a single intraperitoneal injection of sodium pentobarbital (30 mg/kg body weight). The dorsal hair of rats was shaved and disinfected with iodophors, followed by a rinse with 75% (*v/v*) ethanol. Two 10 mm-diameter full thickness wounds were made with a sterile biopsy punch. The rats were maintained under specific-pathogen-free conditions. Diabetic rats were randomly allocated into 2 experiment groups: diabetic treated with ADEE (ADEE group, n = 15) and diabetic without drug treatment (DM group, n = 15), and non-diabetic rats served as normal control (NC group, n = 15). Rats in ADEE group received ADEE 1.1ml/0.2kg body weight once daily by oral gavage, while rats in NC and DM group received 1.1ml/0.2kg body weight distilled water once daily. The dose of ADEE used in rats was 1.2g/kg body weight, which was calculated according to the dose used in patients (0.2g/kg). During the wound healing study, none wound became infected. However, 2 rats in DM group and 1 rat in ADEE group died of the wounding surgery.

### Macroscopic analysis

The healing of rats was monitored by photographing the ulcer every other day with a digital camera until 14 days after wounding. The area of wounds was analyzed with the NIH Image J analyzer by tracing the wound margin with a fine-resolution computer mouse and calculating pixel area. The healing rate was calculated with Wound closure was calculated as Percentage Closed = [(Area on Day 0—Open Area on Final Day)/Area on Day 0]×100, as described previously.

### Immunohistochemistry

The wound tissues with surrounding normal skin of rats were excised and embedded in paraffin after fixing with 10% formaldehyde. Wound sections (4-μm thick) were deparaffinized with xylene and rehydrated through a graded ethanol series. For antigen retrieval, the sections were microwaved in 0.01 mol/L sodium citrate buffer (pH 6.0) at 95°C for 10 minutes, and then cooled to room temperature. Endogenous peroxidase activity was quenched by incubation with 3% hydrogen peroxide for 10 min. The sections were then blocked with 5% BSA for 30 min and then incubated with anti-CD31 (1:200, Santa Cruz Biotechnology, Santa Cruz, CA), followed by incubating with the goat anti-rabbit HRP-conjugated secondary antibodies. The antigen-antibody complex was visualized by incubation with freshly prepared 3, 3’-diaminobenzidine (DAB kit, ZSGB-BIO, Beijing). After counterstained with hematoxylin, the sections were observed with a microscope.

### Aortic ring sprouting assay

For aortic ring sprouting assay [27], the thoracic aortas were harvested from 6-week-old Sprague Dawley rats (n = 4) and transversely cut into 1-mm-thick slices. The aortic rings were placed in 96-well plates coated with 50μl of Matrigel and then sealed in place with an overlay of 20μl of Matrigel. Serum-free DMEM with or without ADEE was added to the wells to a final volume of 100μl. On day 5, microvessel outgrowth was photographed using a phase contrast microscope (Olympus, Tokyo, Japan) and scored from 0 (least positive) to 5 (most positive) in a double-blinded manner. Three independent experiments were performed.

### Cell proliferation assay

EA.hy926 cells were seeded into the 96-well plate at a density of 5×10^3^ cells/well, incubated for 24 h and starved in serum-free media for another 24 h. Proliferation was determined 24h after ADEE or VEGF treatment using the Cell Counting Kit-8 (CCK8; Dojindo Laboratories, Japan) according to the manufacturer’s instructions. Briefly, After 24 h of treatment, the medium was replaced with 110μl DMEM containing 10μl CCK-8 solution. This solution was incubated with the cell cultures at 37°C for 4 h in a humidified atmosphere containing 5% CO_2_. Optical densities of the supernatants were measured at 450 nm with an ELISA spectrophotometer.

### Wound-healing assay

HUVECs were grown to confluence in a 6-well plate and then starved with low-serum (0.5% FBS) media for 8 hours. Subsequently, the cell monolayer was wounded with a sterile plastic pipette tip. After wounding wash the well thoroughly with PBS to remove detached cells and cell debris. The cells were treated with serum-free media containing VEGF or ADEE for 24 hours. The healed area was calculated using Image-Pro Plus 6.0 software by comparing the original images taken immediately after wounding with the images taken after 24 hours incubation with different treatment in the same microscopic field. Those images were obtained using an optical microscope (Olympus).

### Tube formation assay

A 24-well culture plate was coated with Growth factor-reduced Matrigel (250 μl, 10 mg protein/ml, BD). HUVECs were seeded on top of the layer of Matrigel at a density of 2×10^5^ cells/well, followed by the addition of ADEE or VEGF. After incubation at 37°C for 20 h, tube formation was quantified by measuring tube length using Image-Pro Plus 6.0 software from the images taken by a microscope.

### Western blot analysis

Angiogenic effects of ADEE could be a result of its direct action on endothelial cells or through the induction of other genes, such as VEGF, which is a potent angiogenic factor for endothelial cells [28]. So far, PI3K/Akt, eNOS/NO, and ERK1/2 signal pathways have been identified to strongly modulate the angiogenesis process in endothelial cells. Activation of these pathways could directly lead to active proliferation, migration, and tube formation of endothelial cells [29–31]. In order to clarify the mechanisms of ADEE’s angiogenic effects, we detected the levels of VEGF expression and phosphorylated and total ERK1/2, Akt, and eNOS in ADEE stimulated HUVECs through Western blot analysis.

HUVECs treated for indicated time periods was lysed in RIPA buffer. Protein content was measured with BCA Protein Assay Reagent (Pierce, Rockford, IL). Equal amount of protein (30μg) were separated by SDS polyacrylamide gel electrophoresis (PAGE) and transferred onto a polyvinyldifluoride membrane (Millipore) by electroblotting. The membranes were blocked for 2 hours at room temperature with TBST containing 5% non-fat milk, followed by incubation at 4°C overnight with 1:500 diluted anti-VEGF, 1:1000 diluted anti-ERK1/2, phospho-ERK (Thr-202/Tyr-204), Akt, phospho-Akt (Ser473), eNOS and phospho-eNOS (Ser113). After washing three times, the membranes were incubated with the corresponding horseradish peroxidase-conjugated anti-rabbit or anti-mouse antibody for 1 hour at room temperature. Then the membranes were rinsed 3 times and the bands were were detected using an ECL kit (Bio-Rad) and quantified with NIH Image J analyzer.

### Quantitative RT-PCR

Total RNAs were extracted from HUVECs using the Trizol Reagent kit (Invitrogen, CA, U.S.A.). 1μg of total RNA was reverse-transcribed using an RevertAid™ kit (Thermo Fisher Scientific, Waltham, MA). Reverse transcription polymerase chain reaction (RT-PCR) was performed using the CFX96 real-time PCR system (Bio-Rad, USA) with the SYBR Green PCR Kit (Takara, Otsu, Japan) for VEGF-A and GAPDH. Primer sequences are as follows: for VEGF-A 5’ GGG CAG AAT CAT CAC GAA GT 3’ and 5’ ACA CAG GAT GGC TTG AAG AT 3’; for GAPDH 5’ CTC CTC CAC CTT TGA CGC TG 3’ and 5’ TCC TCT TGT GCT CTT GCT GG 3’. GAPDH was used as an internal control. Data were analyzed with 2^-ΔΔCT^ method.

### Nitric oxide (NO) measurement

Diaminofluorescein-FM diacetate (Beyotime Biotechnology, Shanghai, China) was used to measure intracellular NO level [32]. Briefly, 8 h after ADEE treatment, cells were washed twice with serum-free medium and then incubated with 5 μM DAF-FM diacetate for 1 h at 37°C to form the DAF-FM/NO adduct. After removing the excess probe by washing with sterile PBS for three times, the relative levels of intracellular NO were determined by comparing the fluorescence intensity of treated cells with those of untreated cells.

### Statistical analysis

The data are presented as means ± standard error (S.E.) of at least three independent experiments, each performed in triplicate. Statistical analysis were performed using SPSS 16.0 software. One-way analysis of variance (ANOVA) test was used to determine statistical significance. A *P*-value less than 0.05 was considered statistically significant.

## Results

### ADEE improved wound healing in diabetic rats

**Fig 1:**
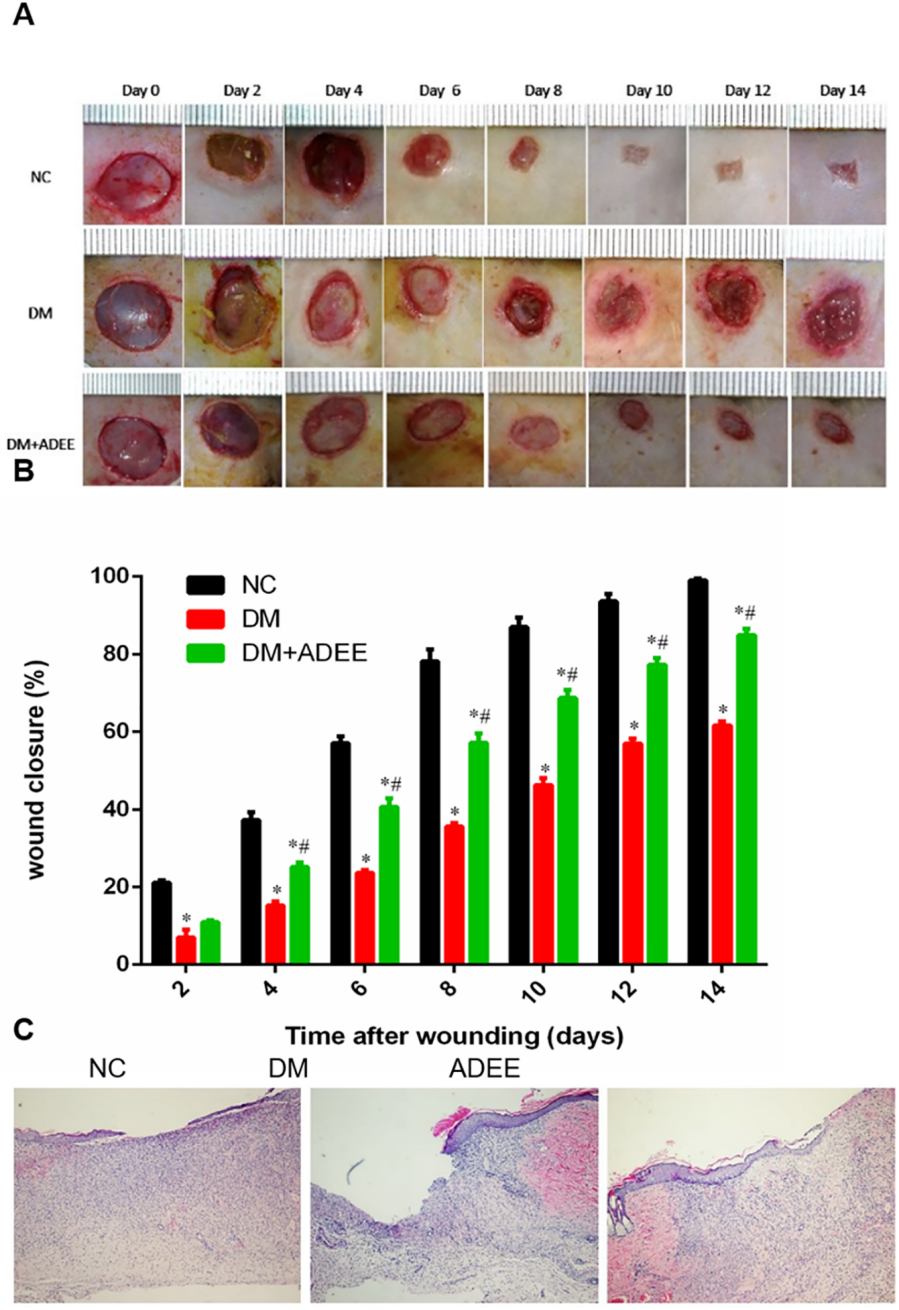
ADEE treatment accelerated wound healing in diabetic rats. Wound closure of 10-mm punch biopsies was monitored for 14 days. (A) Typical photographs of wound healing in each group. (B) Wound closure (mean ± SEM) calculated with the NIH Image J analyzer by tracing the wound margin and calculating pixel area. n = 10. *p<0.05 *versus* NC and ^#^*p*<0.05 *versus* DM. (C) Hematoxylin and eosin (H&E)-stained transverse sections of the wound beds at 8 days showed that ADEE treatment stimulated neovascularization, granulation tissue formation, and epithelium proliferation of diabetic wounds (×100).

STZ-induced diabetic rats were treated with ADEE oral gavage once daily to examine its effects on impaired diabetic wound healing. Excisional wounds of 10-mm diameter were created on the back of normal, diabetic, and ADEE-treated diabetic rats. Healing rate of diabetic rats was significantly slower compared with normal rats. Administration of ADEE accelerated wound closure in diabetic rats since 4 days (Fig 1A and 1B). HE staining of the wound beds showed more granulation tissue formation, neovascularization and proliferative epithelium at 8 days (Fig 1C). These results suggested that ADEE treatment was capable of correcting impaired wound healing and accelerating wound closure in diabetic rats.

### ADEE induces angiogenesis *in Vivo* and *ex Vivo*

**Fig 2:**
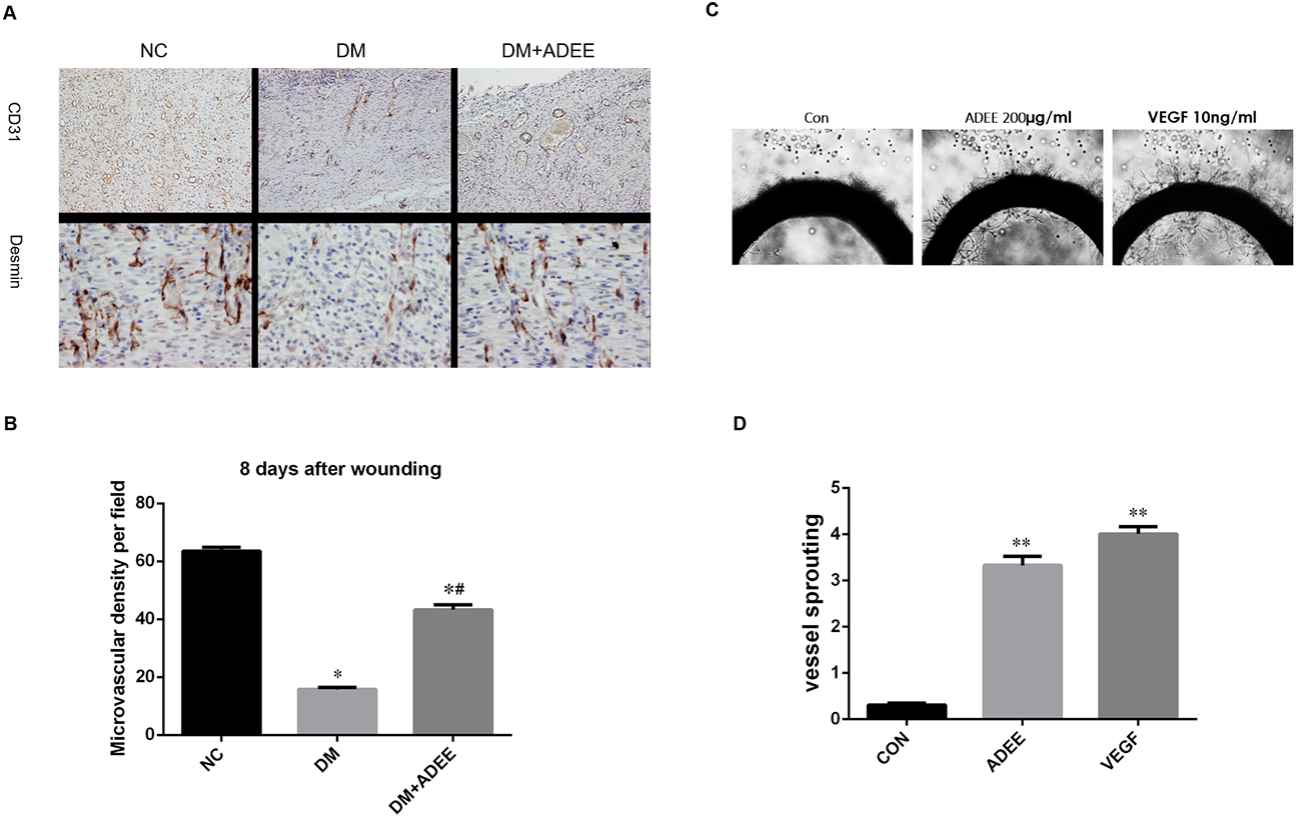
ADEE induces angiogenesis *in vivo* in diabetic wounds and *ex Vivo* in rat aortic ring culture. (A) Representative CD31 and desmin immunostaining sections of NC, DM, and DM+ADEE group on days 8 after wounding, respectively (×100). (B) Five “hot spots” in each specimen in which the CD31 antibody signal was the most intense were chosen and captured. The number of blood vessels was then counted by two investigators who were blinded to the treatment of the rats using the “manual tagging” feature in Image Pro-Plus 6.0 software package. Data are presented as means ± SEM (n = 15). ^*^ *p* < 0.01, compared with NC group, ^#^ *p* < 0.01, compared with DM group. (C) Representative photographs of endothelial cell sprouts growing out of the margin of vessel segments (×50). Thoracic aortas harvested from Sprague-Dawley rats were transversely cut into 1-mm-thick sections. The aortic rings were embedded in Matrigel in 96-well plates and cultured with media containing ADEE (200μg/ml) or VEGF (10 ng/ml) for 5 days. (D) Aortic ring sprouting activity was scored from 0 (least positive) to 5 (most positive) in a double-blinded manner. Data shown are the mean ±S.E.M (n = 6). **p<0.01 *versus* control.

Neovascularization is an essential event in the process of wound healing, which is seriously blunted in diabetes [6, 7]. Therefore, to investigate whether ADEE protected against impaired diabetic wound healing through inducing angiogenesis, we evaluated the neovascularization in the wound tissues on days 8 after wounding by immunostaining endothelial cell marker CD31 and pericyte marker desmin (Fig 2A). The vessel density and pericyte recruitment of untreated diabetic wounds was significantly decreased compared with that of normal wounds. Administration of ADEE significantly increased both the number of newly formed blood vessels of diabetic wounds (*p* < 0.01 as shown in Fig 2B) and pericyte recruitment, indicating more functional blood vessels. To further examine the angiogenic activity of ADEE *ex vivo*, we used rat aortic ring sprouting assay. Treatment with ADEE induced nearly an 11-fold increase in endothelial sprouting from the margin of rat aortic rings compared with control (0.31±0.13 vs. 3.33±0.56) (Fig 2C and 2D). Taken together, these *in vivo* and *ex vivo* results provide explicit evidence that ADEE is able to activate angiogenesis, which contributes to ADEE’s effects in normalizing impaired diabetic wound healing.

### ADEE activates proliferation, migration, and tube formation of HUVECs

**Fig 3:**
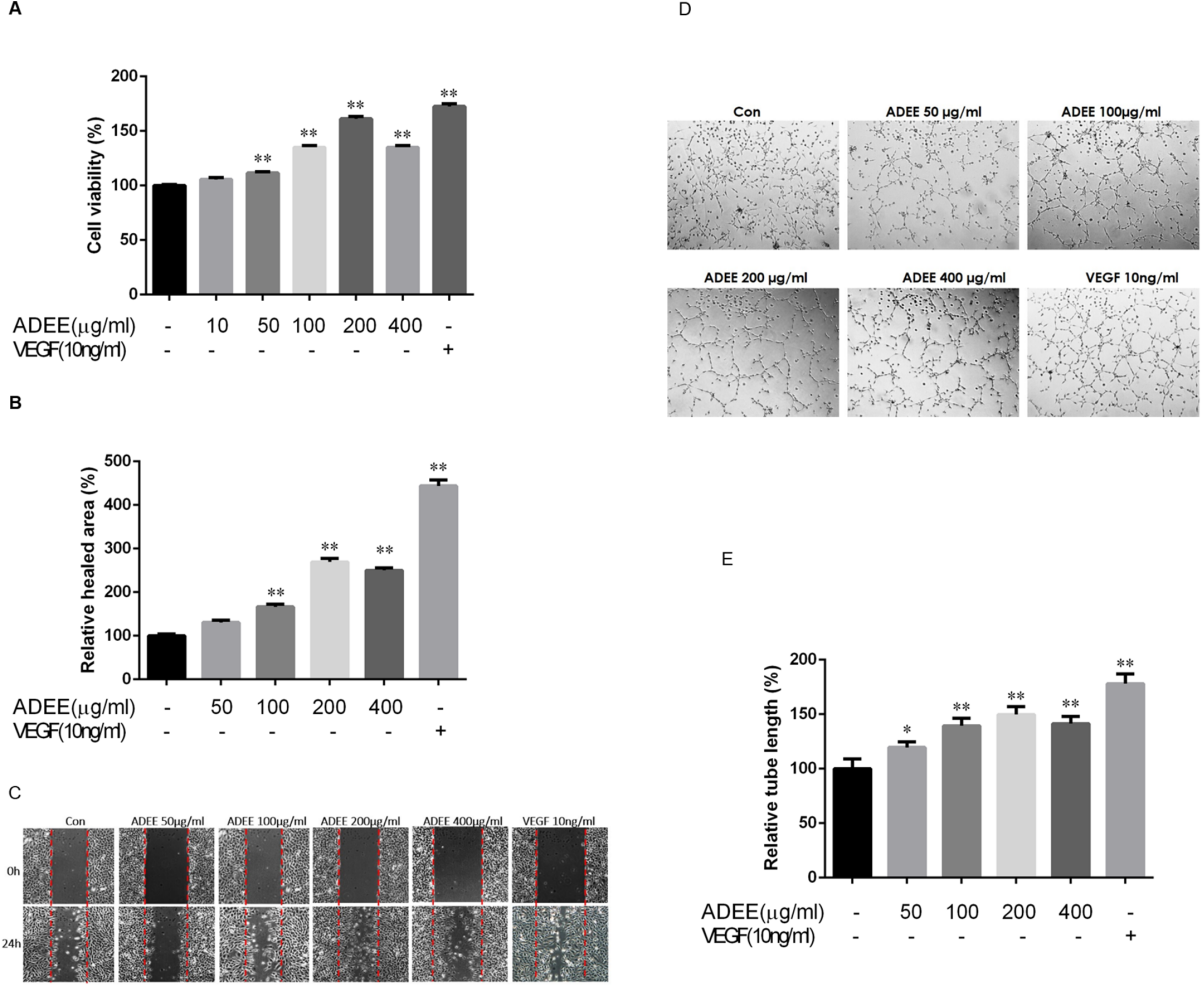
ADEE activates proliferation, migration, and tube formation in HUVECs. (A) After incubation with different concentrations of ADEE (10–400μg/ml) or VEGF (10ng/ml) for 24h, HUVECs proliferation was determined by CCK-8 assay. n = 6. (B, C) Wound healing assay: confluent monolayer HUVECs were wounded by a pipet tip. Before and after 24 hours treatment with ADEE of various concentration or VEGF, photos of the same microscopic field were taken under an inverted phase contrast microscope to calculate the healing area using Image-Pro Plus 6.0 software. Data were expressed as a ratio to control group. n = 10. (D, E) Tube formation of HUVECs cultured on a layer of Matrigel was recorded with an inverted phase contrast microscope after 20h incubation with different treatment. The tube length was quantitated using Image-Pro Plus 6.0 software. Three independent experiments were performed. n = 9. *p<0.05 and **p<0.01 versus control.

Endothelial cell migration, proliferation, and tube formation are crucial steps in angiogenesis. We investigated the role of ADEE in these angiogenic processes *in vitro* using an HUVEC cell line EA.hy926. Treatment with ADEE induced a dose-dependent increase in endothelial cell viability, with a maximal 1.5-fold increase at 200 μg/ml (Fig 3A), comparable to that of VEGF treatment (10 ng/ml). Wound-healing assays showed ADEE stimulated endothelial cell migration in a dose-dependent manner with maximal effects at 200μg/ml (Fig 3B and 3C). To test the effect of ADEE on HUVEC tube formation, we used a Matrigel assay. ADEE increased endothelial tube formation dose-dependently ((Fig 3D and 3E), with the maximal effect at 200μg/ml. These results confirmed that ADEE has powerful angiogenic activity *in vitro*.

### ADEE stimulation of angiogenesis is mediated via the ERK1/2, PI3K/Akt and eNOS/NO signaling pathways

**Fig 4:**
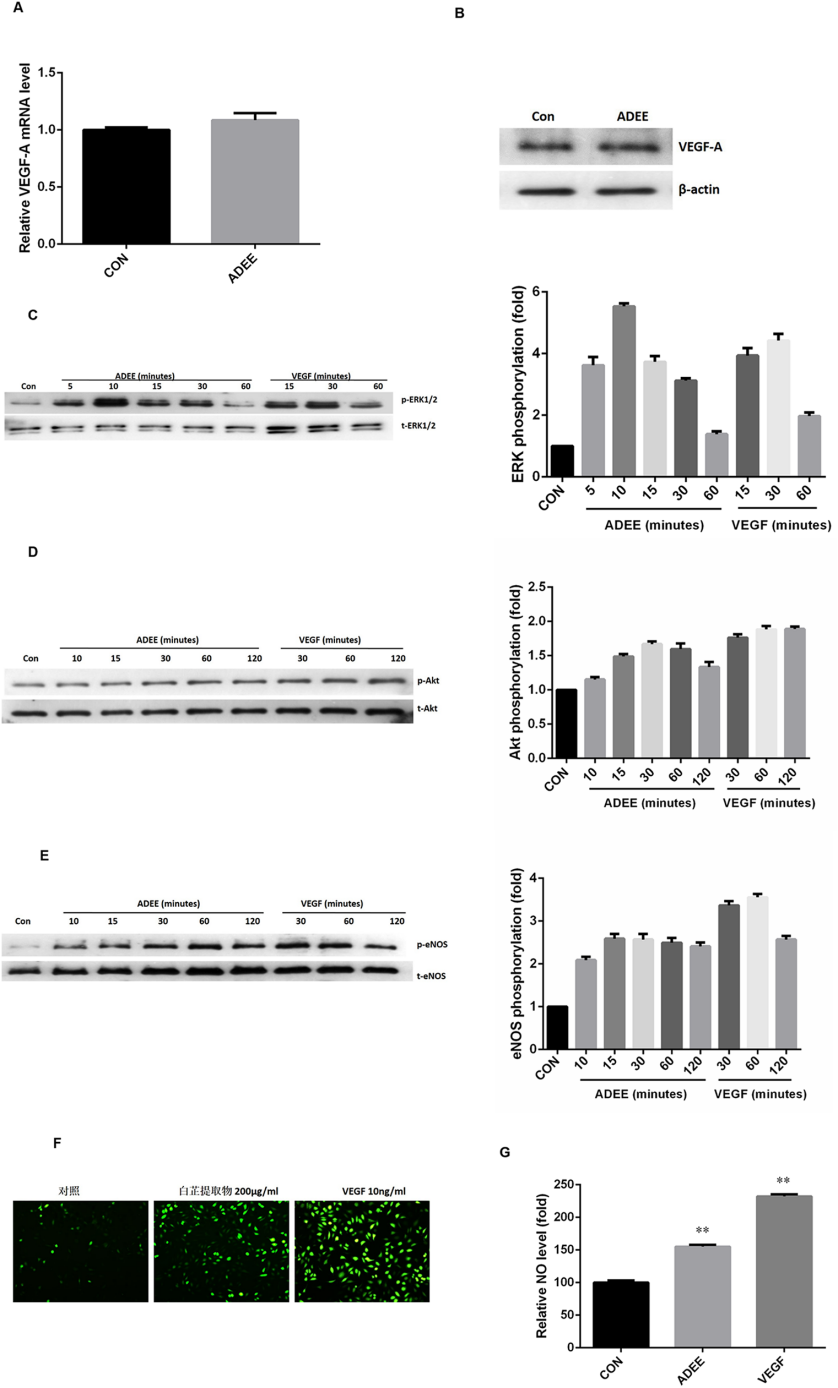
ADEE stimulates the activation of ERK1/2, Akt, eNOS, and NO production in HUVECs. (A, B) HUVECs were incubated with or without ADEE (200 μg/ml) for 6 hours. Expression of VEGF was assessed by real-time PCR (A) and Western blot analysis (B). (C-E) HUVECs were incubated with 200 μg/ml of ADEE or 10ng/ml of VEGF for the indicated time periods. The levels of phosphorylated and total ERK1/2 (C), Akt (D), and eNOS (E) were detected through Western blot analysis. Data were expressed as a ratio to unstimulated control values (CON). (F, G) HUVECs were treated with 200 μg/ml of ADEE or 10 ng/ml of VEGF for 2 hours, and intracellular NO production were determined with a fluorescent microscope. Data were expressed as a ratio to unstimulated control values (CON). **p<0.01 versus control. Experiments were repeated for at least three times.

As stated above, angiogenic effects of ADEE could be a result of either its direct action on endothelial cells or through the induction of other genes, such as VEGF, which is a potent angiogenic factor for endothelial cells [28]. Thus, we examined the expression of VEGF in endothelial cells stimulated with ADEE for 6 hours. However, the mRNA and protein level of VEGF was not affected by ADEE treatment (Fig 4A and 4B), indicating that VEGF induction is not the underlying mechanisms of ADEE-induced angiogenesis. Activation of PI3K/Akt, eNOS/NO, and ERK1/2 signal pathways could directly lead to active proliferation, migration, and tube formation of endothelial cells [29–31]. To test the possibility that ADEE may directly stimulate angiogenesis of endothelial cells, we examined the effect of ADEE on upstream pathways of angiogenesis, including phosphorylation of ERK1/2, Akt, and eNOS as well as NO production. Treatment of HUVECs with ADEE induced ERK1/2 phosphorylation in a time-dependent manner, with maximal activation at 10 minutes, and this intense ERK1/2 activation lasted until 30 minutes (Fig 4C). In addition, ADEE treatment also increased Akt and eNOS phosphorylation time-dependently, with maximal effect at 30 minutes (Fig 4D and 4E). NO, as an important angiogenesis-mediating factor, is induced by angiogenic factors through activating Akt-dependent eNOS phosphorylation. Consistent with the eNOS phosphorylation, ADEE elevated NO production in HUVECs compared with control (Fig 4F and 4G). VEGF was used as a positive control for its ability to activate angiogenesis through inducing ERK1/2, Akt, and eNOS phosphorylation as well as NO production [33]. The ability of ADEE activating Akt and eNOS pathways is comparable to VEGF, and ADEE induced even stronger phosphorylation of ERK1/2 compared with VEGF, while VEGF induced more NO production (Fig 4C–4G). These results indicate that ADEE induced angiogenesis of HUVECs may involve signaling pathways of ERK1/2, Akt, and eNOS as well as NO production.

**Fig 5:**
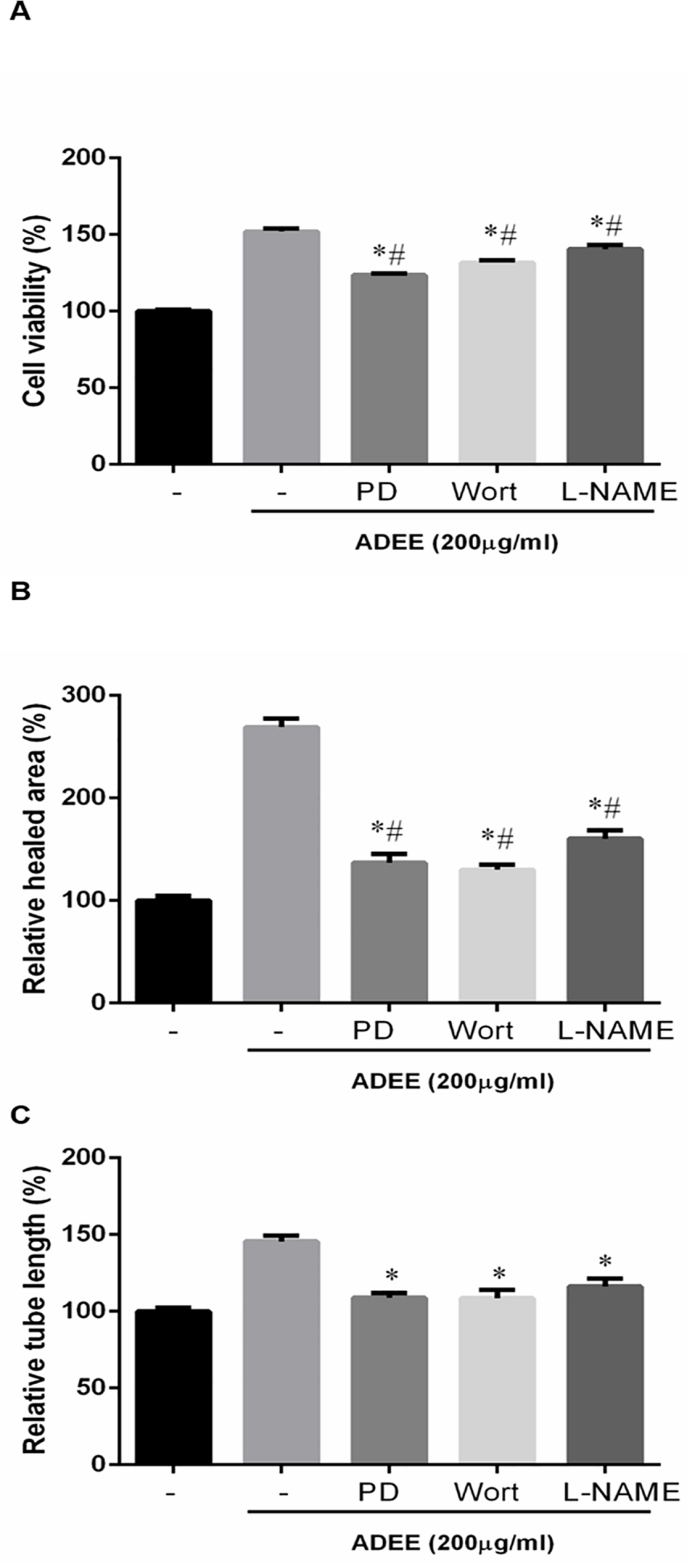
ADEE induced proliferation, migration, and tube formation of HUVECs were inhibited by MEK, PI3K, and eNOS inhibitor. (A) Before treated with 200 μg/ml ADEE, HUVECs were incubated for 30 min with or without 10 μM PD98059 (PD), 100 nM wortmannin (Wort), or 2mM L-NG-nitroarginine methyl ester (L-NAME). The proliferation of HUVECs was determined by CCK-8 assay. (B) Confluent monolayer HUVECs were pretreated for 30 min with or without 10 μM PD98059, 100 nM wortmannin, or 2mM L-NAME, wounded by a pipet tip, and then stimulated with 200 μg/ml ADEE. Healed area was determined as described in Fig 3. (C) Tube formation of HUVECs induced by ADEE in the presence of 10 μM PD98059, 100 nM wortmannin, or 2mM L-NAME was recorded. *p<0.05 versus positive control. #*p*<0.05 versus blank control. Experiments were repeated for at least three times.

Next, we examined the involvement of Akt, ERK, and eNOS in ADEE-mediated angiogenesis by blocking those pathways with the MEK inhibitor PD98059, the PI3K inhibitor wortmannin, and the NOS inhibitor L-NG-nitroarginine methyl ester (L-NAME), respectively. ADEE-mediated increases in endothelial cell proliferation and migration were partially suppressed by wortmannin and PD98059, and L-NAME (Fig 5A and 5B). However, tube formation induced by ADEE-was nearly abolished by all three inhibitors (Fig 5C). These results suggest that the activation of ERK1/2, PI3K/Akt, and eNOS/NO functions as important signal mediators in ADEE -induced angiogenic process.

### ADEE accelerates inflammation resolution and improves healing quality of diabetic wounds

**Fig 6:**
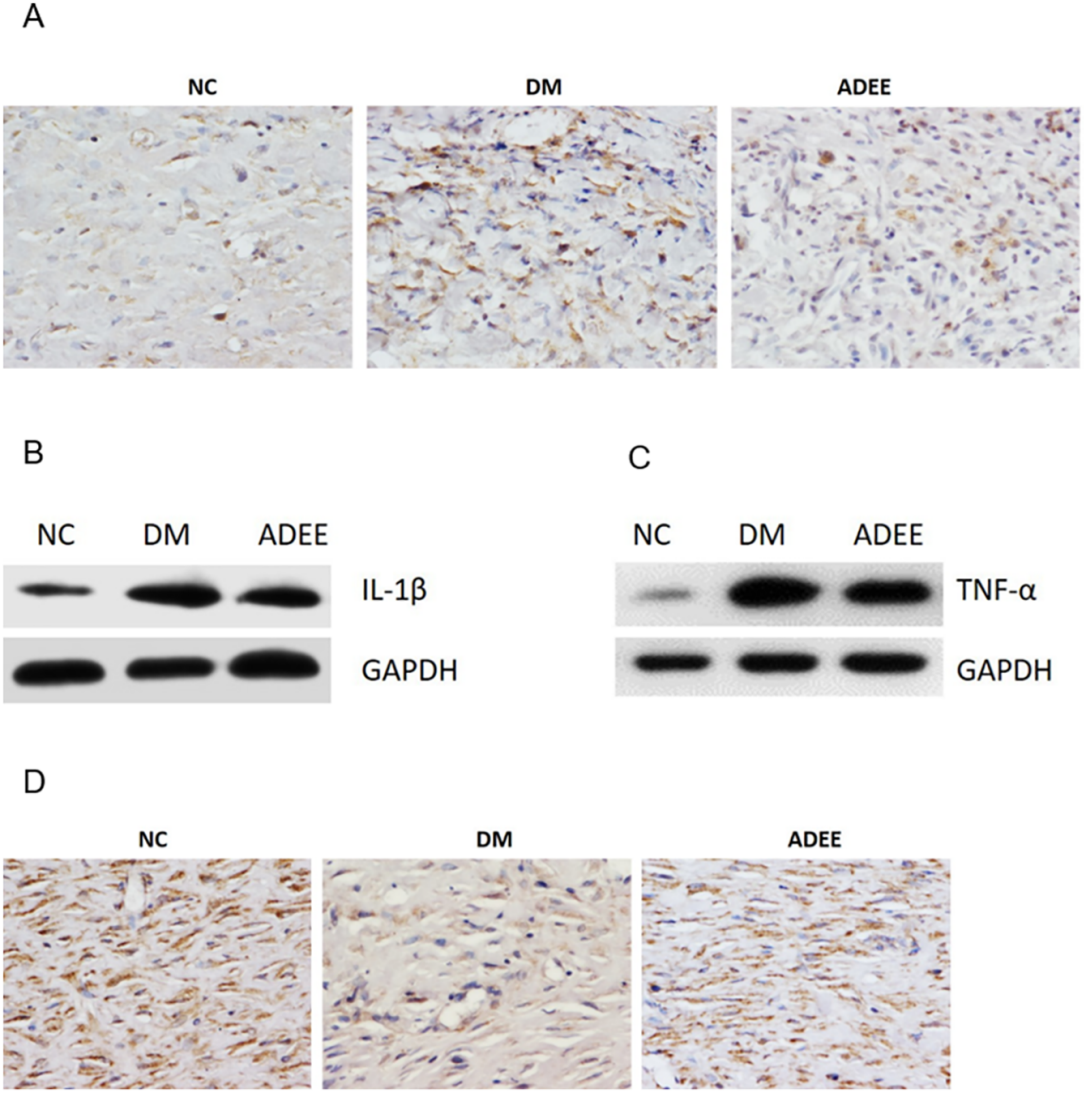
ADEE accelerates inflammation resolution and improves healing quality of diabetic wounds. (A) CD68 staining of wounds from 14 days postwounding. (B, C) Western blotting assay of IL-1β and TNF-α expression of wounds from 14 days postwounding. (D) Immunostaining of collagen Ⅰ of wounds from 14 days postwounding.

Delayed inflammation resolution, another hallmark of diabetic wounds, is associated with bad healing outcome, for example, delayed wound closure and poor healing quality [34]. Increased and prolonged infiltration of inflammatory cells release high levels of inflammatory cytokines, which suppress migration and function of other important repair cells, such as fibroblast. Fibroblast produces extracellular matrix, for example collagen Ⅰ, which are responsible for the tensile strength of the wound. To evaluate the effects of ADEE on inflammation resolution and healing quality of diabetic wounds, we performed immunohistochemistry and western blot using wounds from 14 days postwounding. Compared with NC group, rats in DM group had more CD68-positive (macrophage marker) cells and higher levels of IL-1β and TNF-α, and ADEE treatment reversed the increased levels of infiltrated inflammatory cells and inflammatory cytokines (Fig 6A–6C). Besides, the deposition of collagen Ⅰ was greatly improved by ADEE administration (Fig 6D), suggesting better function of fibroblasts.

Our study showed that ADEE treatment decreased the number of CD68-positive cells and the expression of IL-1β and TNF-α, and improved the deposition of extracellular matrix by fibroblast.

## Discussion

Many factors contribute to the impaired wound healing in diabetes. Angiogenesis is a pivotal process in wound repair, however, it is seriously blunted in diabetic wounds [6].

Non-healing wounds leads to amputation, and even death, posing a serious threat to the health of diabetic patients [35].

Medicinal herbs have long been used as wound healing agents around the world. In traditional Chinese medicine, the boiled roots of *Angelica* species has been applied internally or externally, alone or combined with other herbs extracts, to wounds to speed healing. Recent studies have also proved the wound healing effects of some species of Angelica. For example, *Angelica sinensis* has been found to stimulate wound healing and increase the strength of the healed wounds [16]. Another *Angelica* species named *Angelica dahurica*, was recorded in many Chinese medical classic as an herb that has the promoting granulation, anti-inflammatory, and analgesic effects [36]. Our previous studies[25] showed that ethanolic extract of TLXDS, a TCM formula composed of *Angelica Dahurica* and three other herbs (*Angelica sinensis*, *Astragalus membranaceus* and *thorns of Gleditsia sinensis*), accelerated wound healing in STZ-induced diabetic rats through increasing angiogenesis and reducing inflammation. To further explore the active components and underlying mechanisms of TLXDS, we examined the therapeutical effects of *Angelica dahurica* in TLXDS on diabetic wound healing.

The present study showed that *Angelica Dahurica* ethanolic extract (ADEE) had a remarkable effect on angiogenesis both *in vivo* and *in vitro*. ADEE significantly improved healing of diabetic rats through increasing angiogenesis, in concert with its direct angiogenic activity in HUVECs by stimulating proliferation, migration, and tube formation. Our results suggest that ADEE can be utilized as a potential therapeutic herbal medicine for impaired wound healing in diabetes, and perhaps other angiogenesis-related diabetic complications, e.g. coronary heart disease.

PI3K/Akt, eNOS/NO, and ERK1/2 signal pathways are critical modulators in the process of angiogenesis [29–31]. The activation of PI3K/ Akt phosphorylates eNOS at serine 1177, leading to the elevation of NO production in endothelial cells [37]. Mounting evidence have shown that NO is a powerful angiogenic mediator and can induce endothelial cell proliferation, migration, and tube formation [31, 38, 39].The angiogenesis and wound healing of eNOS deficient mice was significantly delayed compared with wild type mice [40]. Moreover, NO is a major vasodilative substance released by the endothelium [41]. The hallmark of endothelial dysfunction in diabetes is impaired endothelium-dependent vasodilation, which is associated with decreased NO production [42]. A defect in NO production has also been proposed as a major contributor to atherosclerosis [43] which is an important pathogeny for diabetic foot. As noted above, imperatorin was reported to exert its actions of vasodilation and protection against cardiac hypertrophy through inducing eNOS phosphorylation and NO production [20, 21]. In line with previous studies, we demonstrated that ADEE-induced endothelial cell proliferation, migration, and tube formation was accompanied by activation of PI3K/Akt/eNOS signal pathway and elevated NO production in endothelial cells. These effects were similar to those observed following VEGF treatment. Blocking the activation of Akt and eNOS by wortmannin and L-NAME significantly decreased ADEE-induced endothelial cell migration and tube formation. These findings indicate that the PI3K/Akt/eNOS pathway plays an important role in ADEE-stimulated angiogenesis. Considering the vasodilative and cardiac protective effects of NO, when used in diabetic patients who developed endothelial dysfunction and atherosclerosis because of NO defection, ADEE may provide extra protection against impaired wound repair, not limited to increasing angiogenensis.

ERK1/2 pathway has been implicated in endothelial cell proliferation [44], survival [45]. Another way ERK signaling is thought to stimulate angiogenesis is by promoting endothelial cell motility [46]. As shown in this study, ADEE activated ERK1/2 pathway and selectively blocking ERK1/2 using PD98059 inhibited ADEE induced angiogenesis. These findings supported that ERK1/2 activation also has a distinct role in ADEE-induced angiogenesis.

Moreover, ADEE induces angiogenesis without affecting VEGF expression, suggesting ADEE may function as a direct angiogenic modulator in endothelial cells.

Prolonged inflammation is another important feature of diabetic wounds. Hyperglycemia and oxidative stress associated with diabetes sustain prolonged influx of inflammatory cells through inducing excessive proinflammatory cytokines (IL-1β, TNF-α, etc.) production [47, 48]. Persisting inflammatory cells together with impaired angiogenesis result in reduced extracellular matrix deposition such as collagen Ⅰ. The present study showed that administration of ADEE significantly accelerated inflammation resolution by decreasing the expression of inflammatory cytokines, such as IL-1β and TNF-α, and thereby reducing the macrophages abundance in diabetic wounds. And this effect is accompanied by increased deposition of collagen Ⅰ, suggesting better proliferation and function of fibroblasts.

Growing evidences have proved the effectiveness of TCM in the management of diabetes and its complications [49–51]. Herbal medicine are of great therapeutic values waiting researchers to explore and to elucidate the underlying mechanisms. Our present study demonstrated that the ethanolic extract of *Angelica Dahurica*—ADEE has a beneficial role in improving diabetes-impaired wound healing via its direct effects on neovascularization and inflammation resolution, and the angiogenic role of ADEE is exerted through pathways involving ERK1/2 and Akt/eNOS phosphorylation and NO production.

## Supporting information

S1 Fig HPLC chromatograms of ADEE. (A) HPLC chromatogram of Standard substance of imperatorin detected at 300 nm. The blue arrow showed imperatorin. (B) HPLC chromatogram of ADEE detected at 300 nm. The blue arrow showed imperatorin. (C) Imperatorin contents in ADEE. The data were represented as mean ± SEM.
(DOCX)
