# Supplementary material for: Angelica Dahurica ethanolic extract improves impaired wound healing by activating angiogenesis in diabetes
Source: PLoS One. 2017 May 24;12(5):e0177862. doi: 10.1371/journal.pone.0177862 (PMC5443501; doi:10.1371/journal.pone.0177862)
Supplement: S1 Fig — (A) HPLC chromatogram of Standard substance of imperatorin detected at 300 nm. The blue arrow showed imperatorin. (B) HPLC chromatogram of ADEE detected at 300 nm. The blue arrow showed imperatorin. (C) Imperatorin contents in ADEE. The data were represented as mean ± SEM. (DOCX) [file pone.0177862.s001.docx]

To determine the chromatographic profiles of ADEE, the extract was analyzed by High Performance Liquid Chromatography (HPLC) analysis at 300 nm. HPLC analysis was performed on an Agilent 1100 liquid chromatograph system to determine the component in ADEE. The compounds were monitored at 300 nm using a Agilent C8 HPLC Column (250×4.6 mm, 5 µm). The column was operated at 20°C, and the injection volume was 10 µL. The mobile phase consisted of methanol and water (51:49) at a flow rate of 1.0 mL/min. Previous reports have shown that the main constituent of *Angelica dahurica* was imperatorin; therefore, we analyzed imperatorin using HPLC at 300nm as a standard control. Imperatorin were detected in the ADEE by comparing the retention of the chromatographic images with that of standard (S1 Fig A-C).

**A**

**
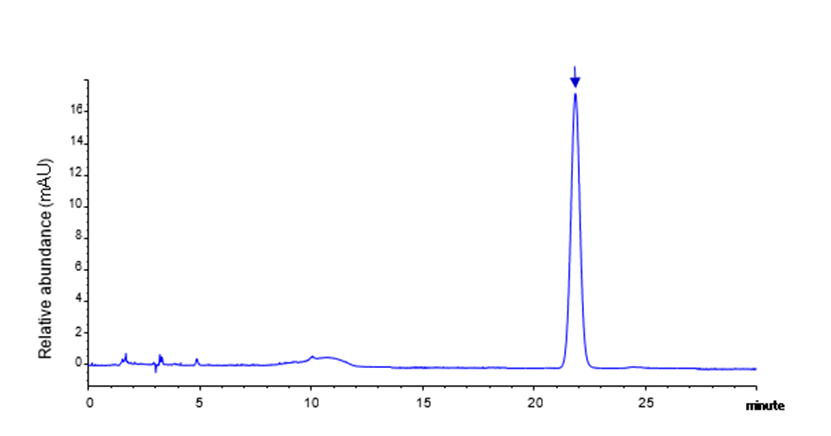
**

**B**


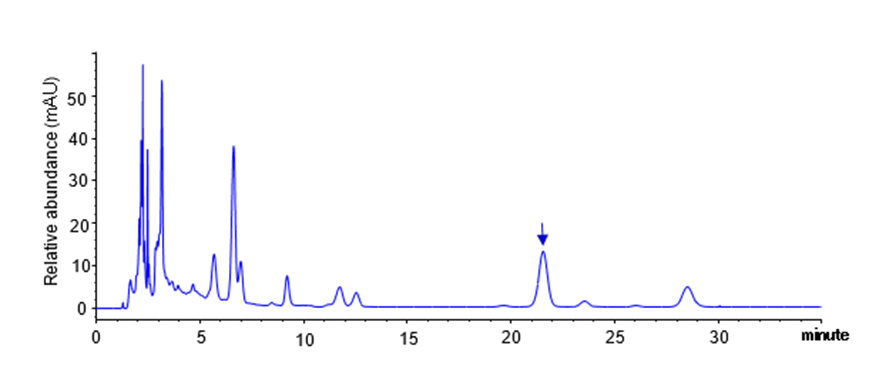


C





S1 Fig. **HPLC chromatograms of ADEE.** (A) HPLC chromatogram of Standard substance of imperatorin detected at 300 nm. The blue arrow showed imperatorin. (B) HPLC chromatogram of ADEE detected at 300 nm. The blue arrow showed imperatorin. (C) imperatorin contents in ADEE. The data were represented as mean ± SEM.
